# Surgery for hip fractures: Does surgical delay affect outcomes?

**DOI:** 10.4103/0019-5413.73660

**Published:** 2011

**Authors:** Nicole Simunovic, PJ Devereaux, Mohit Bhandari

**Affiliations:** Department of Clinical Epidemiology & Biostatistics, Division of Orthopaedic Surgery, McMaster University, Ontario L8N 3Z5, Canada; 1 Department of Clinical Epidemiology & Biostatistics and Department of Medicine, McMaster University, Ontario L8N 3Z5, Canada

**Keywords:** Hip fractures, surgical timing, mortality

## Abstract

Hip fractures are associated with a high rate of mortality and profound temporary and sometimes permanent impairment of quality of life. Current guidelines indicate that surgeons should perform surgery for a hip fracture within 24 hours of injury because earlier surgery is associated with better functional outcome and lower rates of perioperative complications and mortality. Proponents of early treatment argue that this approach minimizes the length of time a patient is confined to bed rest, thereby reducing the risk for associated complications, such as pressure sores, deep vein thrombosis, and urinary tract infections. Those favoring delaying surgery beyond the guideline recommendations believe that this approach is required to medically optimize patients, and therefore decrease the risk for perioperative complications. Further challenges to resolving this debate is the lack of an accepted definition of what should constitute an “unacceptable delay” for hip fracture surgery and the fact that outcomes associated with surgical delay are based on observational data alone (i.e., not randomized controlled trials). The effect of preoperative timing on mortality and other patient-important outcomes across various age groups remains controversial and warrants a large randomized controlled trial to offer clear insights into the effects associated with early versus delayed surgery among hip fracture patients.

## INTRODUCTION

Among elderly patients, hip fractures are associated with an in-hospital mortality rate of 7–14%^1^,^2^ and profound temporary and sometimes permanent impairment of independence and quality of life.^3^ As the elderly population increases, the number of hip fractures globally is expected to exceed 7–21 million annually over the next 40–50 years^4^ with significant costs to health care systems.^5^–^7^

Current guidelines^8^ recommend that surgeons perform hip fracture surgery within 24 hours of injury as observational studies suggest earlier surgery is associated with better functional outcome and lower rates of nonunion, shorter hospital stays and duration of pain, and lower rates of complications and mortality.^1^,^9^–^14^ Even though data suggesting a delay to surgery of more than 24 hours may not unequivocally impact mortality, these guidelines recommend early surgery on the basis that elderly (>50-60 years of age) hip fracture patients “are at risk of complications, and on compassionate grounds merit early intervention.”^15^

Proponents of early treatment argue that this approach minimizes the length of time a patient is confined to bed rest, thereby reducing the risk of associated complications, such as pressure sores, deep vein thrombosis, pneumonia, and urinary tract infections.^11^,^16^ However, those favoring a delay in timing of surgery believe that it provides sufficient opportunity to medically optimize patients, and therefore decrease the risk for perioperative complications.^11^ Further challenges to resolve the debate is the lack of an accepted definition of early surgery.^17^ Uncertainty exists about whether 24, 48, 72 hours or more should be considered an “unacceptable delay” for hip fracture surgery.

This paper reviews the literature surrounding the effect of surgical timing for the treatment of hip fractures on commonly reported outcomes, including mortality, postoperative complications, and length of hospital stay.

## METHODS

Current evidence regarding the timing of hip fracture surgery is based on the observational data. Observational studies are prone to selection, performance, attrition, and detection bias. Well-done prospective observational studies remain generally less biased than retrospective studies because the predictor variable is measured before the outcome; thus establishing a time sequence of events and preventing predictor measurements from being influenced by knowledge of the outcome.^18^ The prospective approach also allows investigators to measure multiple exposures and events more completely and accurately than is possible retrospectively.^18^ Based on a prior systematic search by the authors, and a recently published review on operative delay for hip fracture patients,^19^ approximately 65% of studies addressing this issue are retrospective, and therefore may be subject to confounding and biased ascertainment of outcomes.

Observational study results may be adjusted for different confounding variables using regression analysis. In simple terms, regression analytic methods predict outcome on the basis of risk factors for patients on each treatment or exposure and then evaluates whether the observed outcome for patients for a given variable (e.g., early versus delayed surgery) is better than that predicted. One could argue that a significant contributing, if not the main factor affecting adverse outcomes in patients undergoing delayed surgery is that these patients tend to be sicker on admission, and are therefore more likely to, first, require greater time to surgery for preoperative testing and treatment, and second, experience an adverse event when compared to those that undergo immediate surgery.^20^,^21^ Indeed, several studies have shown that when patient function and comorbidities were adjusted for in the analyses, in-hospital mortality was no longer associated with the preoperative interval when compared to the unadjusted results.^9^,^11^,^22^,^23^ Adjusted data can help to determine if operative delay itself is a cause of poor outcomes, or if it merely reflects a higher risk group of patients who require more time for medical stabilization. The authors recently conducted a systematic review of prospective studies on surgical timing for the outcome of mortality in hip fracture patients (unpublished). Based on a pooled estimate of five studies (*n*=4208 patients) that computed adjusted odds or hazard ratios for mortality at 30 days,^23^ 6 months,^11^ and 1 year^24^–^26^ (721 total deaths) by means of a multivariate logistic regression model or multivariate Cox proportional hazards model, earlier surgery was associated with a 19% risk reduction in all-cause mortality (relative risk 0.81; 95% confidence interval, 0.68–0.96, *P*=0.01 and *I*^2=^0%).

The most effective way to reduce confounding is with a randomized trial. Randomization of large numbers of patients avoids systematic bias by producing groups that are balanced in terms of both known and unknown prognostic factors. Ethical considerations make it difficult to conduct a randomized, controlled trial where patients are deliberately delayed to surgery.^27^,^28^ We believe, however, that it is reasonable to compare patients in an accelerated treatment arm (e.g., within 12–24 hours) versus routine care (i.e., whenever surgery occurs). Study designs that minimize bias are needed to provide a definitive answer with regard to the effect of earlier surgery among patients admitted with a hip fracture.

## REASONS FOR DELAY

The most common reasons for operative delay include the unavailability of the operating room and/or surgical personnel (administrative), and investigation and stabilization of the patient’s preoperative medical condition (medical-related)^17^,^23^,^24^,^26^,^29^–^38^ It is possible that there is a differential effect for those patients delayed for administrative reasons alone compared to those delayed for the optimization of acute medical conditions consequent to their fractured hip.

### Mortality

In a retrospective, unadjusted analysis of 406 patients with proximal femoral fractures, Kenzora *et al*^39^ found a higher 1-year mortality rate after operative repair on the first hospital day compared with the second through fifth hospital days (34% versus 5.8%, *P*<0.0001). However, they also reported that a large number of medically unfit patients underwent earlier surgery. Sicker patients may benefit from a delay in order to optimize their medical condition and including these patients in the early surgery group may have diluted the true effect of postponing surgery.^2^ For example, Zagrodnick and Kaufner^40^ noted a lower in-hospital mortality rate with preoperative stabilization of medical conditions. A prospective study examining a subgroup of 60 acutely ill, hip fracture patients showed a reduction in mortality with surgery delayed more than 24 hours.^41^ However, these analyses and observations are not sensitive or powerful enough to detect the effect of early and delayed surgery on unhealthy patients alone. Further, other studies have shown that less healthy patients may still benefit from surgery within 24 hours.^42^,^43^

In 778 medically fit patients, Bredahl *et al*.^44^ found that surgery performed within 12 hours significantly reduced unadjusted mortality rates at 5 months to 1 year compared to surgery performed after 12 hours. Other retrospective and prospective studies have shown a significant decrease in 1 year mortality rates in medically fit patients that undergo surgery within 24 hours^17^,^24^,^28^,^30^,^42^,^43^,^45^,^46^ and 48 hours.^35^,^47^–^49^When potential confounding preoperative factors are taken into account, such as pre-existing chronic conditions, age, and mental health status, this effect may not be as large or no longer significant. Zuckerman *et al*.^26^ prospectively studied 367 patients and showed a higher mortality rate during the first year in those patients that underwent surgery later than 72 hours after admission (hazard ratio 1.80, 95% confidence interval 1.03–3.16). The association persisted after controlling for age, gender, and number and severity of pre-existing health conditions (hazard ratio 1.76, 95% confidence interval 1.00–3.10). When Orosz *et al*.^11^ controlled for additional factors such as prefracture residence and mobility, delirium on admission, fracture type, day and time of admission, and history of specific chronic diseases across 1178 patients (17.5% mortality at 6 months), surgery performed within 24 hours was no longer associated with improved mortality (adjusted hazard ratio 0.75, 95% confidence interval 0.52–1.08 versus unadjusted hazard ratio 0.68; 95% confidence interval 0.48–0.97). Other studies have shown no significant association between early or delayed surgery and adjusted mortality rates.^9^,^22^,^23^,^31^,^50^–^52^

Indeed, prospective adjusted results for the effect of surgical timing on mortality outcomes are generally borderline or nonsignificant. Methodological limitations, including lack of sufficient statistical power and inadequate adjustment for known and potentially unknown preoperative factors, have contributed to the lack of a relationship between surgical delay and outcome in many of these studies. Weller *et al*^2^ estimate that over 5000 subjects would be required to detect an odds ratio of 1.2 (80% power; α=0.05) favoring early surgery. Even more patients would be required if adjustments for confounding were to be made^2^ because small studies, including those discussed above, are at substantial risk of producing invalid results as a result of having so few events and patients relative to the number of predictors considered in the analyses. A systematic review on the effect of preoperative timing on mortality, which included 257,367 patients across 16 prospective and retrospective studies, found that a surgical delay of more than 48 hours was associated with increased mortality in hip fracture patients, but noted that “potential residual confounding factors in observational studies may limit definitive conclusions.”^19^

The current evidence suggests that while surgical delay of more than 24 hours may not unequivocally impact mortality, there is no theoretical benefit for healthier patients to wait for surgery. Rather, there is the potential for increased complications and poor outcome.^2^ In the case of medically unfit patients, this effect is less clear.

## POSTOPERATIVE COMPLICATIONS

Surgical timing does not appear to have a significant effect on the number of complications patients may experience after surgery.^26^,^32^,^44^,^46^,^47^,^50^,^53^ Yet some studies have shown a significant association between surgical delay (i.e., >24 hours) and the increased incidence of pressure ulcers^9^,^25^,^27^,^34^,^54^ and avascular necrosis,^55^ both complications consistent with extended bed rest. Two prospective studies that adjusted for patients’ preoperative health status, age, and gender found a significant^27^ and nonsignificant association^56^ between time to surgery and a patient’s return to independent living status. Where the type of surgery has been shown to affect hip fracture patient outcomes,^57^ Al-Ani *et al*. still found a significantly improved ability of patients undergoing earlier surgery to return to independent living even after adjustment for treatment modality, pre-fracture living status, and walking ability.^27^

In a prospective cohort study of 1206 patients, those who underwent surgery within 24 hours had significantly fewer days of severe pain.^11^ Pain causes a stress reaction and subsequent insulin resistance to accelerate the process of muscle loss and weakness,^27^ thereby delaying patient rehabilitation and increasing the risk of delirium.^58^ However, most of these studies are flawed by heterogeneity and a retrospective design. In the absence of a randomized prospective study comparing delayed and expeditious surgery, it is difficult to know whether surgical delay adversely affects outcomes directly or whether delay in surgery is simply a reflection of underlying morbidities that adversely affect these complications.

## DURATION OF HOSPITAL STAY

Regardless of the cut-off for delay (e.g., 24, 48, 72 hours) early surgical treatment of a hip fracture injury is associated with a shorter hospital stay based on both unadjusted^17^,^34^,^35^,^44^,^53^,^59^–^61^and adjusted analyses.^11^,^27^,^45^,^50^ For most studies, as the operative delay increased, so did the mean length of hospital stay.

It is expected that the longer a patient is required to wait for surgery, the longer they are in the hospital due to the preoperative wait alone. And while early surgery appears to have a large significant effect on reducing the length of stay, it is difficult to establish whether this effect is maintained over and above the preoperative interval. Future studies should calculate and report on the postoperative length of stay in relation to operative timing to resolve this issue.

## THE ECONOMIC BURDEN OF SURGICAL DELAY

Health care resources incurred for hip fracture patients can include initial hospitalization, rehospitalization, rehabilitation, chronic care, home care, long-term care, and informal care. Costs are substantially higher for patients who do not return to the community and require long-term care or sustain another hip fracture.^7^ In Canada, the cost of hip fractures is $650 million annually, and is expected to rise to $2.4 billion^7^ based on a projected number of 88,124 hip fracture patients by 2041.^62^ The estimated lifetime cost for all hip fractures in the United States in 1997 likely exceeded $20 billion.^63^ In the United Kingdom, direct hospital costs alone were estimated to be $125 million in 2003.^5^

Prompt surgical intervention may not only avoid unnecessary discomfort for the patient and facilitate early mobilization and rehabilitation, but also reduce health care costs. Shabat *et al*.^64^ studied the economic effects of delay to surgery in hip fractures and concluded that spending more resources to perform surgery within 48 hours of admission is more cost-effective than delaying surgery past 72 hours.

## METHODS FOR MINIMIZING SURGICAL DELAY

The management of hip fractures requires complex yet cohesive care from presentation to the emergency unit, through to the departments of radiology, anesthetics, orthopedic surgery, medicine, and rehabilitation. Techniques to expedite preoperative care can shorten operative delays, especially for those patients that have been medically cleared for surgery. For example, a recent systematic review as well as a prospective study of 116 patients found that dedicated trauma coordinators or hospitalists have been shown to be effective at fast-tracking patients with hip fractures to surgery by organizing operating room lists, perioperative care, securing hospital beds, and acting as a liaison with the radiology department and porting services.^29^,^65^ A retrospective analysis of 139 patients found that a dedicated trauma operating room not only reduced the time to dynamic hip screw and closed femoral nailing procedures, but also allowed more of these surgeries to be performed during daytime hours, which may reduce postoperative complications.^66^

However, other evidence suggests that in efficient systems, hospital management variables may not significantly affect patient mortality and morbidity. The Scottish Hip Fracture Audit^31^ collected data relating to 18,817 hip fracture patients and analyzed, through multiple logistic regression models, which factors were potentially associated with postoperative mortality. Significant factors included increased age, male gender, extracapsular fractures, and poor prefracture health and function. Variables that could be modified by preoperative medical interventions, such as surgeon or anesthesiologist expertise and time to surgery, had no significant relationship with postoperative mortality assessed at 30 or 120 days.^31^

## CONCLUSION

The current evidence for optimal surgical timing is entirely observational and often conflicting for the outcomes of mortality, most postoperative complications, length of hospital stay, and return to living status. Unadjusted analyses are certainly confounded, and residual confounding may be responsible for apparent effects in adjusted analyses in these studies. Further, an acceptable time for delay has yet to be established. Clear evidence is needed before we can justify displacing other surgical patients to facilitate hip fracture surgeries. A definitive answer to this issue requires a large randomized controlled trial to evaluate the effect of earlier surgery among patients admitted with a hip fracture.
